# Symptom appraisal and healthcare-seeking for symptoms suggestive of colorectal cancer: a qualitative study

**DOI:** 10.1136/bmjopen-2015-008448

**Published:** 2015-10-09

**Authors:** N Hall, L Birt, J Banks, J Emery, K Mills, M Johnson, G P Rubin, W Hamilton, F M Walter

**Affiliations:** 1 School of Medicine, Pharmacy and Health, Durham University, Durham, UK; 2 Department of Public Health & Primary Care, University of Cambridge, Cambridge, UK; 3 School of Social & Community Medicine, University of Bristol, Bristol, UK; 4 Department of General Practice, University of Melbourne, Carlton, Victoria, Australia; 5 Lay member, Cambridge, UK; 6 University of Exeter Medical School, University of Exeter, Exeter, UK

**Keywords:** MEDICAL HISTORY, PRIMARY CARE, QUALITATIVE RESEARCH

## Abstract

**Objectives:**

Timely diagnosis of colorectal cancer is important to improve survival. This study explored symptom appraisal and help-seeking among patients referred to specialist services with symptoms of colorectal cancer.

**Design:**

Qualitative in-depth interview study.

**Setting and participants:**

Participants were recruited on referral to gastroenterology clinics (North East and East of England); interviews were conducted soon after referral. We purposively sampled participants to ensure a range of accounts in terms of age, sex, diagnosis and geographical location.

**Methods:**

Data collection and analysis were underpinned by the Model of Pathways to Treatment. Framework analysis was used to explore the data within and across cases, focusing on patient beliefs and experiences, disease factors and healthcare influences.

**Results:**

40 participants were interviewed (aged 43–87 years, 17 women, 18 diagnosed with colorectal cancer). Patients diagnosed with and without colorectal cancer had similar symptom pathways. We found a range of interacting and often competing biopsychosocial, contextual and cultural influences on the way in which people recognised, interpreted and acted on their symptoms. People attempted to ‘maintain normality’ through finding benign explanations for their symptoms. Bodily changes were appraised within the context of usual bowel patterns, comorbidities and life events, and decisions to seek help were made in relation to expectations about the course of symptoms. The ‘private nature’ of colorectal cancer symptoms could affect both their identification and discussions with others including healthcare professionals. Within the context of the National Health Service, people needed to legitimise appropriate use of healthcare services and avoid being thought of as wasting doctors’ time.

**Conclusions:**

Findings provide guidance for awareness campaigns on reducing stigma around appraising and discussing bowel movements, and the importance of intermittent and non-specific symptoms. Altering perceptions about the appropriate use of health services could have a beneficial effect on time to presentation.

**Strengths and limitations of this study**

- We believe this study is the first to compare the appraisal and help-seeking experiences of patients with symptoms of colorectal cancer (such as rectal bleeding, a change in bowel habit and abdominal pain) between people subsequently diagnosed with colorectal cancer and people diagnosed with other non-cancer conditions; we were unable to identify any differences.
- The study was guided by the Aarhus statement recommendations on improving design and reporting of studies on early cancer diagnosis.
- Risks of recall bias and post hoc rationalisation were reduced by recruiting at the time of referral to specialist care, and interviewing patients before or close to diagnosis.
- The ‘private nature’ of colorectal symptoms is a novel finding, and contributes to our understanding of why some people may not present in a timely way with symptoms suspicious of colorectal cancer. Symptoms that can no longer be kept private may prompt help-seeking.

## Introduction

Colorectal cancer (CRC) is the second most common cause of cancer-related death in Europe.^1^ ^2^ While the recent implementation of population-based CRC screening programmes across many countries will help to reduce CRC mortality,^3^ most CRCs are still detected symptomatically,^4^ and prompt symptomatic diagnosis remains a priority. The relationship between time to diagnosis and survival is complex;^5^ however, later stage at diagnosis is thought to be one of the factors responsible for the lower CRC survival rates in the UK and Denmark in relation to other comparable Western countries.^6^ The time from symptom onset to healthcare-seeking has been estimated to represent the greatest proportion of the total time to diagnosis.^7^ In order to develop interventions to optimise early cancer diagnosis and improving treatment outcomes, it is therefore essential to understand influences on symptom appraisal and healthcare-seeking.

There remains limited understanding of why people choose to seek care for symptoms associated with CRC when they do, and how the influence of contributory factors varies throughout the diagnostic pathway.^8^ Although there are generic influences on the way in which people interpret and act on their symptoms, there may also be influences specific to the site of the cancer.^9^ ^10^ The time taken to recognise the potentially serious nature of symptoms is the most commonly reported factor contributing to consultation behaviour for symptoms of CRC and delayed diagnosis.^11^ However, warning signs for CRC, such as persistent change in bowel habit and rectal bleeding, are common,^12^ and are not a sign of serious illness for most people who experience them.^13–15^

To date, research in this area has largely focused on healthcare-seeking behaviour for a particular symptom such as rectal bleeding, or has utilised retrospective accounts of patients with CRC whose diagnosis may colour their narrative.^16^ In contrast, this study aimed to investigate symptom appraisal and help-seeking among patients with a range of symptoms suggestive of CRC as they were referred from primary care to secondary care for further investigation.

## Methods

### Participants and procedures

We conducted semistructured in-depth qualitative interviews with individual participants aged ≥40 years who had been referred with symptoms suggestive of CRC to five gastroenterology clinics at hospitals in the North East and East of England. This study was nested within a larger prospective study (the SYMPTOM study) that aimed to identify factors associated with later presentation to primary care and later stage at diagnosis for people with symptoms suspicious of lung, colorectal and pancreatic cancer.^17^ ^18^ Participants were purposively sampled from the responders who had agreed to be interviewed, to ensure diversity in terms of age, gender, geographical location and diagnoses. The semistructured interview schedule focused on contributing factors, intervals, events and processes within the appraisal and help-seeking intervals of the Model of Pathways to Treatment.^19^ ^20^ Open-ended questions allowed additional topics to be explored.

Interviews lasted between 40 and 90 min and were undertaken by NH, LB and KM between 2011 and 2013. They were scheduled as close to the referral to secondary care as possible to minimise recall bias (median period from referral to interview 10 weeks, range 4 weeks to 5 months), and written informed consent was gained from all participants. A specifically developed calendar landmarking instrument was employed throughout the interviews to assist with clarifying the sequence of events and the time periods between them.^21^ Interviews continued until no new themes could be identified, indicating saturation of data. All interviews were recorded and transcribed verbatim. Secondary care medical records were searched postinterview for final diagnoses.

**Figure 1:**
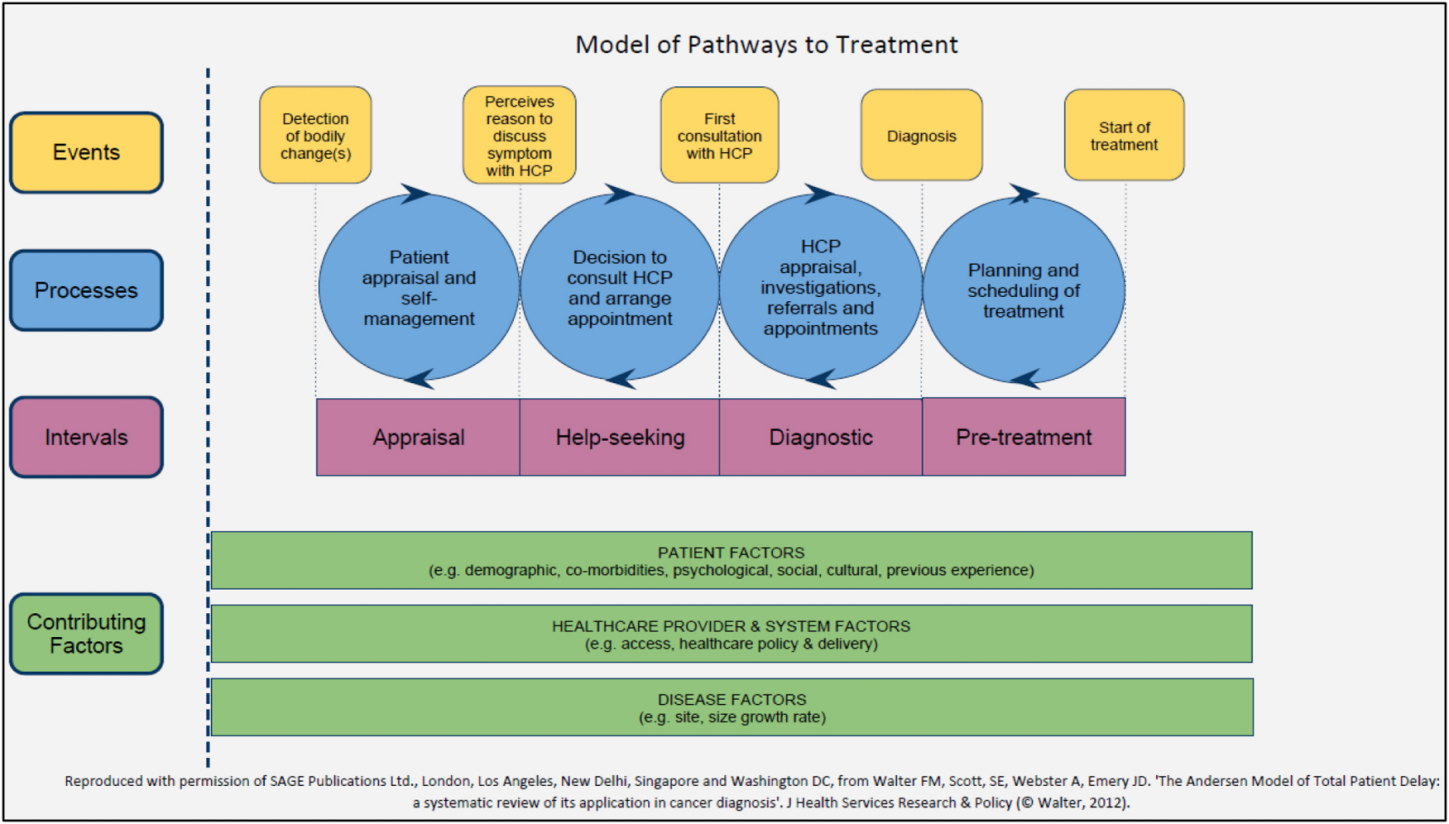
Model of Pathways to Treatment.^18^

The Model of Pathways to Treatment^19^ ^20^ was used as a theoretical framework to guide both data collection and analysis (figure 1) as recommended in the Aarhus statement for studies of early cancer diagnosis research.^22^ It defines the events and processes that can occur within four overlapping intervals (appraisal, help-seeking, diagnostic and pretreatment) from the initial detection of a bodily change to the start of treatment. It also highlights the patient, healthcare and disease factors that can influence these processes and is underpinned by established psychological theories, including the Common Sense Model (CSM) of Illness Self-regulation 23 and Social Cognitive Theory.^24^

### Data analysis

Symptom appraisal and help-seeking decisions were explored within and across cases using an approach based on framework analysis.^25^ Analysis followed five stages: familiarisation, identification of a thematic framework, indexing, charting, mapping and interpretation. An initial thematic framework incorporated key concepts and themes from the interviews alongside the processes, intervals and contributing factors from the Model of Pathways to Treatment. All transcripts were indexed by either NH or LB using the analytic framework and 10% of transcripts were indexed by both researchers, with good inter-rater reliability. The data from each interview were summarised and charted into a coding matrix, which was used to aid comparison within and across cases. This allowed the range of the identified phenomena to be mapped within each category and the identification of patterns and associations between themes and across cases. In addition, a lay representative (MJ) independently identified themes from a selection of transcripts which were compared with the existing analysis through discussion with NH and LB. Data management was supported by NVivo9 software.

All supporting quotations are identified by sex, age group, CRC or non-cancer (NC) diagnosis, and total time to presentation (TTP) from first noticing a bodily change to their first consultation with a healthcare provider.

## Findings

### Participant characteristics

Interviews were completed with 40 participants (table 1). Eighteen participants were diagnosed with CRC and 22 with non-cancer diagnoses; 12 were unaware of their diagnosis at the time of their interview.

**Table 1:** Participant characteristics

		Diagnosis	
	Total n=40	CRC cancer N=18	Other diagnoses* N=22
Location of interview			
East Anglia	17	8	9
North East	23	10	13
Female	18	8	10
Male	22	10	12
Age			
Mean years (range)	63 (43–87)	65 (49–85)	62 (43–87)
40–59	13	4	9
60–79	23	12	11
80+	4	2	2
Ethnicity			
White British	37	17	20
Other	3	1	2
Educational qualifications†			
Higher education	15	8	7
Post 16 or vocational	15	5	10
Secondary school	2	1	1
None	7	4	3
IMD quintiles			
1 (least deprived)	14	6	8
2	9	5	4
3	4	1	3
4	5	2	3
5 (most deprived)	8	4	4
Referral type			
2 week wait/urgent	23	14	9
Emergency	3	1	2
Routine	14	3	11
GP appointments made before referral			
0	3	1	2
1	24	11	13
2	7	2	5
3 or more	6	4	2
Median weeks from 1st reported symptom to presentation (range)	12 (0–100)	13 (1–77)	11 (0–100)
Percentage of waiting >6 weeks from noticing first reported symptom to presentation	71	81	63
Percentage of waiting >6 weeks from first noticing trigger symptom to presentation	29	16	42
Comorbidities			
IBS	2	0	2
Diabetes	3	2	1
Arthritis	11	4	7
Lung/heart disease	13	6	7
None	15	7	8

*Polyps (5), diverticulitis (5), haemorrhoids (4), IBS (4), fissure (1), angiodysplasia (1), constipation (1), anaemia (1), spirochetosis (1), colitis (1).

†1 missing data item.

CRC, colorectal cancer; IBS, irritable bowel syndrome; IMD, Index of Multiple Deprivation; GP, general practitioner.

### Routes to help-seeking

All participants described noticing a relevant bodily change before their initial contact with a HCP. Their first noticed bodily change did not always prompt a appointment with a HCP, and referral did not always take place after the first presentation to a HCP (usually their general practitioner (GP)); a third of participants (n=13) described repeated appointments before referral. Although many presented to their GP with a bowel-related symptom of concern, other routes to diagnosis were also described, such as via routine appointments for other conditions, emergency appointments for acute symptoms and follow-up of other investigation results.

Participants described variable patterns in the nature of their symptoms, presentation and routes to referral regardless of their final diagnosis. Their symptoms included: blood noticed on toilet paper, in the toilet bowl or with stools; looser or more frequent stools, diarrhoea, constipation, or other changes to the colour, consistency or shape of stools; loss of bowel control; wind and bloating; abdominal pain, discomfort or cramps; anaemia, fatigue, shortness of breath and weight loss. Some reported symptoms they had not initially associated with a ‘bowel condition’, including persistent cough, swollen ankles, vomiting, sickness and fever.

The Model of Pathways to Treatment recognises the dynamic, rather than linear, nature of movement through the pathway, and this was reflected in the accounts of our participants. In some cases with a sudden onset such as an alarming rectal bleed, events and processes had occurred simultaneously and were difficult to disentangle. Even with the aid of a calendar landmark tool, attributing a specific time point to key events was particularly difficult for participants with vague, intermittent, gradual or non-specific changes or in the presence of existing long-standing symptoms or comorbidities.

I had the symptoms all last year but I mean I could well have had them longer than that but they wouldn't, you know, it wasn't like they were every day or all day or anything and they were very mild … (Female, 65–69, CRC, TTP: 40–52 weeks)

Regardless of their severity, intermittent symptoms were sometimes experienced as a series of separate appraisal episodes ‘linked’ in hindsight, rather than one period of continual symptom monitoring:

So I think throughout, like I had periods where I wasn't feeling well, then I'd feel better in between…it wasn't one sort of constant period of not feeling well really … (Female, 44–49, CRC, TTP: 13–26 weeks)

As a third of our participants consulted more than once before being referred to a specialist, our findings also reflect ongoing symptom appraisal and repeated healthcare-seeking beyond the first consultation with a HCP.

### Main themes

**Figure 2:**
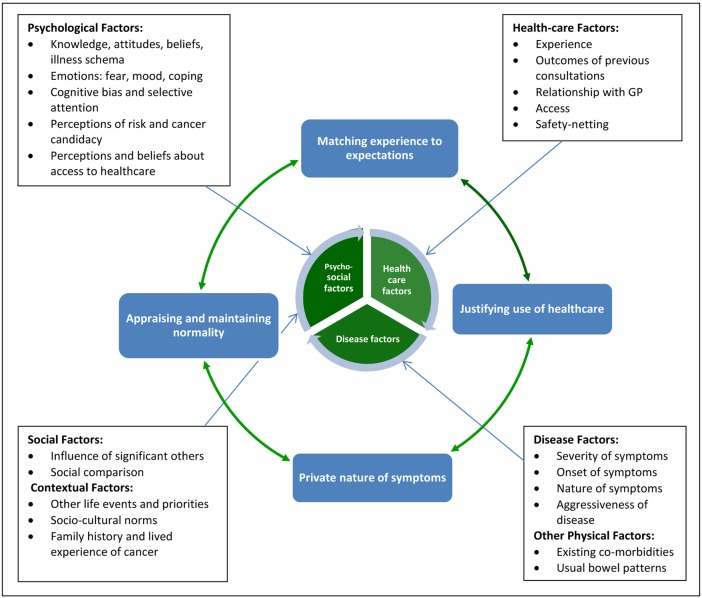
Factors affecting symptom appraisal and help-seeking among people with colorectal cancer symptoms (GP, general practitioner).

We identified four main interdependent and overlapping themes that operated across the appraisal and help-seeking intervals: ‘appraising and maintaining normality’; ‘matching experiences to expectations’; ‘private nature of symptoms’, and ‘justifying healthcare use’. These themes are presented as different lenses through which to understand some of the ways symptom specific, psychosocial and cultural factors interact and influence the processes that combined to form participants’ ‘help-seeking journeys’ (figure 2). Importantly, we did not find any substantive differences between people diagnosed with cancer and other conditions.

### Appraising and maintaining normality

Participants reflected on bodily changes that they subsequently recognised as symptoms. The detection of a ‘change’, as well as its subsequent appraisal, took place in relation to a fairly wide spectrum of what was considered to be ‘normal’ for individuals. For example, a change in bowel habit was compared to usual or expected patterns in terms of timing, stool consistency and frequency.

The first symptoms I noticed was probably a year, 18 months ago, looser stools than would be normal but lasting for … it wasn't diarrhoea but it lasted for more than the normal two or three days … (Male, 65–69, CRC, TTP: 53+ weeks)

These were more likely to be noticed quickly if participants had a previously regular bowel habit.

I always used to go to the loo, to pass a stool first thing in the morning, well about an hour after breakfast and then I've noticed that actually I need to pass a stool later in the day as well. (Female, 70–74, NC, TTP: 13–26 weeks)

Vague or non-specific changes, such as fatigue, were particularly difficult to detect and were sometimes only recognised as ‘unusual’ with hindsight. The following participant reported not having noticed any change at all.

I've just always been like this and maybe I've always been a bit anaemic so I don't really notice the difference. (Female, 50–54, CRC, TTP: 0–2 weeks)

The term ‘symptom’ reflects *‘*the transition from a bodily change to something perceived as abnormal’.^20^ Changes were more likely to be recognised as abnormal when they were severe or of sudden onset, became repeated or other changes developed, persisted beyond an expected time, interfered with daily activities, or became a focus of attention or concern by others. Changes were often initially ‘normalised’ as signs of ageing, stress or diet, or attributed to ‘stomach bugs’ or food poisoning.

Maintaining normality was often achieved by dietary changes, self-medication or seeking reassurance from family or friends. Seeking healthcare was more likely when the emotional or physical impact of symptoms could no longer be managed or had become a threat to normal daily life.

If you can carry on, you don't bother to go, it's too much aggravation, it's only when things start getting in the way of what you want to do … that one bothers to think about it as a reportable condition. (Male, 75–79, CRC, TTP: 13–26 weeks)

You just have to carry on and soldier on for everybody else. So I couldn't find the time for myself, and because it got so bad I had to go the GP, otherwise I would have still carried on. (Female, 45–49, NC, TTP: 13–26 weeks)

For a few participants, maintaining normality involved a period of adaptation to severe and debilitating symptoms, even faecal incontinence. For these people, a decision to seek healthcare often required a significant trigger event.

### Matching experience to expectations

On the basis of people’s understanding of the cause of their symptoms, they had expectations about the likely course and resolution of their symptoms. CRC was not usually considered as a possible cause by those who had presented with ‘non-bowel’ symptoms such as vomiting or symptoms of anaemia. CRC was also not considered by those who had attributed their bleeding to haemorrhoids, had been reassured about bleeding by a HCP in the past (including recent consultations), or had reasons to suspect other serious illness such as colitis. The presence of blood was the symptom most likely to prompt a consideration of cancer or the need to seek healthcare, but this was not universal.

It was the bleeding, that was the significant thing ‘cos I mean that is not normal, whereas mild tummy ache or mild bloating, you know, I think if you ask every woman of my age they would all confess to that. (Female, 65–69, CRC, TTP: 9–12 weeks)

Participants were more likely to consider CRC when there was a strong family or personal history of cancer. Expectations about the significance of symptoms were influenced by cultural beliefs, family and friends.

So how can I be ill if my weight doesn't change?, because my mam said “if you're ill you lose weight. (Female 50–54, NC, TTP: 40–52 weeks)

For those who had considered cancer as a potential cause of their symptoms, negative bowel cancer screening test results, an absence of blood or pain, weight loss or ‘not feeling ill’ were perceived as reassuring signs.

Even then I wasn't thinking of cancer, I'd never associated that sort of symptom (faecal incontinence) with cancer, to me it always meant that you would have some form of pain or you would lose weight rapidly … and feel ill which I had none of those symptoms at all. (Male, 65–69, CRC, TTP: 53+ weeks)

Many participants expressed concern about the possibility of a cancer diagnosis, but this was usually more prominent after their initial consultation with their GP. Fear of cancer did not seem to be a barrier to seeking healthcare.

People's explanations for their symptoms were often transient and varied over time; some were described merely as passing or fleeting thoughts. When symptoms did not resolve as expected, alternative causes were considered. Knowledge and awareness of cancer signs, although important, were not sufficient, nor always necessary, for a decision to seek healthcare promptly. A decision to seek healthcare was triggered when symptoms no longer matched what was expected in terms of frequency, duration, severity or impact on daily life or when alternative explanations could not be found.

### The private nature of symptoms

Since colorectal symptoms are mostly experienced privately, many participants felt that opportunities for comparing symptoms with others were more limited than with other more ‘visible’ symptoms such as a persistent cough; this could make their significance harder for people to assess. We also found that sociocultural norms about the private nature of colorectal symptoms could reduce opportunities for detection and influence symptom appraisal.

Me personally, the way I was brought up, one, you don't talk about downstairs, and two, you don't look at downstairs. So, when people ask me questions like “Is it in the stool?” or anything like that, I tend not to look, I do now but I didn't then. (Female, 50–54, NC, TTP: 40–52 weeks)

Nonetheless, many people reported speaking to partners, family, friends and work colleagues about their symptoms. When symptoms usually contained within the home could no longer be controlled or ‘hidden’, the embarrassment and impact on daily life could act as a trigger for seeking healthcare.

I've been having that wind problem. (Laughs) … It's horrible, really embarrassing. And if I should be out and I get this sort of urge to go to the toilet, I have to go otherwise I've pooed myself … Very embarrassing. (Female, 70–74 NC, TTP: 13–26 weeks)

Many participants had been aware that an appointment with their doctor about their colorectal symptoms might involve a rectal examination, and some were aware of the possibility of further invasive investigations such as colonoscopy. Few felt this had affected their decision to seek healthcare. Concern about being perceived to be wasting the doctor's time was more likely to be reported. Nevertheless, when embarrassment or other concerns about invasive investigations were present, these could present a significant barrier to consultation.

I did think about it a few times before I actually went (sighs) but I don't know why, nothing to do with thinking about cancer, it was just the embarrassment of knowing that I might have to have somebody's finger pushed up your bum for an examination … that was probably the thing that put me off going more than anything in the first instance was the embarrassment of that sort of thing … I don't suppose anybody likes, whether it's a doctor or not, playing around what you consider as your private parts and that, it's not the most pleasant of experiences. (Male, 65–69, CRC, TTP: 53+ weeks)

Potential embarrassment about an intrusive test such as colonoscopy could be outweighed by the concern caused by the symptoms and the need for reassurance. Some suggested that embarrassment around invasive procedures reduced with age.

I mean, I'm at an age now where it doesn't matter to me who I see, I don't mind people fiddling around with me whatever gender they are … You lose all this, kind of, this, sort of dignity and stuff as the years go by. (Male, 80–84, NC, TTP: 5–8 weeks).

### Justifying the use of healthcare

Participants’ reactions to symptoms depended on symptom attributions, as well as on culturally defined and implicit beliefs about the appropriate use of healthcare*.* These beliefs had the most impact during the decision to seek healthcare, yet also influenced continued symptom appraisal and self-management. Concerns about being perceived to ‘waste the doctor's time’ and using publicly funded healthcare resources inconsiderately were commonly reported. Waiting for a symptom to recur, persist or worsen allowed a ‘justification’ of the use of healthcare resources. Participants rarely talked about the time taken to present to a HCP in terms of intentional delay and were more likely to report that they had sought care as soon as they realised this was necessary or they felt justified in doing so.

I went online to book an appointment and the first one that came up was three weeks ahead, and I thought that's fine by me … if they (symptoms) haven't gone away then I'll feel justified in bothering her really. (Female, 65–69, CRC, TTP: 5–8 weeks)

For those with a cancer concern, the importance of ‘catching it early’ and having a good relationship with the HCP were used to legitimise healthcare-seeking. Bleeding or pain were seen to be more legitimate reasons for consulting than non-specific symptoms regardless of whether they were associated with a cancer concern or not. A recent negative bowel screening test meant that consulting was seen as less appropriate by some participants.

It wasn't sufficient really to go to the doctor, I didn't think so because I'd already had two of the bowel cancer tests that come through the post. (Male 65–69, CRC, TTP: 53+ weeks)

A national bowel cancer awareness campaign took part during our study period (January–March 2012). Advertisements encouraged people with blood in their stools or looser stools for 3 weeks to present to a GP and to not be embarrassed about their symptoms. Not everyone who had seen the campaign associated the message with their symptoms, although it helped to trigger and legitimise repeated help-seeking for one woman who presented to her GP three times before she was referred.

People said if you have it for three weeks, any sign of blood, get yourself to doctor, doesn't matter how embarrassed, just say it to your doctor. This is what I did, I followed the campaign … and I went back straightaway because I know it's not right. (Female, 50–54, CRC, TTP: 13–26 weeks)

Not all the participants believed that their symptoms warranted a specific appointment with their GP and some participants mentioned their colorectal symptoms only during follow-up or routine appointments.

I was talking to him [GP] and then I thought, “Yeah, I'll tell him, I'll ask him about it … Well yeah. I thought, “It's silly not to, I'm here.” You know, it's more practical to ask him about it, so I did. (Female, 60–64, CRC, TTP: 9–12 weeks)

Although for some participants talking to family and friends had an important influence on their decision-making, this was not the case for everyone. Encouragement from significant others could act as both a prompt and a means of legitimising healthcare-seeking.

My youngest daughter said you can't, she's a bit on the bossy side, “Don't leave it Mam, go.” And so I probably would have left it a bit longer if she hadn't have said. (Female, 65–69, NC, TTP: 0–2 week)

Reassurance could have the opposite effect and resulted in delayed healthcare-seeking for the following participant.

I choose to see my doctor but when I talk to a few friends, they say “oh we all have constipation here” … so I just ignore it. (Female, 50–54, CRC, TTP: 13–26 weeks)

For those who had presented to their GP more than once, advice from the doctor about appropriate repeat visits, when provided, had been an important facilitator for timely repeat consultations, influencing symptom monitoring and legitimising the continued use of services.

## Discussion

### Principal findings

This study has confirmed that recognising the significance of common symptoms associated with CRC is far from straightforward, and is often a challenge for patients prior to seeking healthcare.^8^ Our findings highlight a range of interacting and often competing biopsychosocial, contextual and cultural influences on the way in which people recognise, interpret and act on their symptoms of CRC. Importantly, we did not find any substantive difference between people diagnosed with cancer and other conditions. Bodily changes are appraised within the context of usual bowel patterns, comorbidities and life events, and people attempt to maintain normality through finding benign explanations for their symptoms. Decisions to seek help often occur only when their expectations about the course of symptoms are not met. Our most novel finding relates to the private nature of CRC symptoms which may affect their identification as well as discussion with others including HCPs; once it becomes impossible to conceal their private nature, this may also prompt help-seeking. Within the context of the publicly funded National Health Service, people needed to legitimise appropriate use of healthcare services and avoid being seen to be wasting doctors’ time.

### Strengths and weaknesses of this study

This qualitative study was embedded in a large prospective cohort study, which allowed us to purposively sample from a wide range of perspectives and sociodemographic backgrounds. This is one of the first such studies to include a reasonably large sample of patients with similar symptoms, not all of whom had a final diagnosis of CRC. Importantly, participants were recruited either before diagnosis or shortly afterwards, reducing potential recall bias. Both data collection and analysis were underpinned by the Model of Pathways to Treatment which has a strong psychological theoretical basis.^20^ We also used a calendar landmark instrument which is designed to improve the chronology of the symptom pathways reported by participants.^21^ Analysis was performed by a group of experienced qualitative researchers from different disciplinary backgrounds; in addition, we included a patient representative to provide a unique perspective to the analysis and interpretation. There are some limitations to our study. Despite our efforts to reduce recall bias, there will probably be some post hoc rationalisation of patients’ experiences, particularly for those who knew their cancer diagnosis at the time of the interview. Although we were able to include three participants from ethnic minority groups, this was not enough to make conclusions regarding specific cultural influences.

### Comparison with existing literature

Previous research in a range of cancers has identified normalisation as a way of coping with, and minimising, symptoms.^26^ Our findings also reflect the social and cultural context in which these processes occur, mirroring research based on Alonzo's concept of containment.^27^ Our participants’ accounts demonstrate that although knowledge and awareness of the signs of CRC can have an important influence, they are not always necessary, nor sufficient, for timely healthcare-seeking. Indeed, a mismatch between expectations and experienced symptoms can influence beliefs about the perceived seriousness and the need to seek healthcare. Similar findings came from a recent Danish study where most patients recently diagnosed with CRC had not attributed their initial symptoms to cancer, and only rectal bleeding was likely to make people consider cancer.^28^ Our study also confirmed previous research in pancreatic cancer that intermittent symptoms were acted on only when a pattern was identified, there was a change in their frequency or nature, or they became associated with additional symptoms.^29^ Many of the heuristics from established psychological research known to influence the interpretation of symptoms were apparent in our data and are reflected across our second theme ‘matching experience with expectations’.^20^ For example, a lack of bleeding, pain or weight loss, intermittent symptoms and not feeling ‘ill’ confirmed ‘non-worrying’ attributions.

The influence of the private nature of colorectal symptoms provides a novel insight into possible reasons for later help-seeking. This impacted on whether people observed subtle changes in their stools, as well as on whether these were discussed with close others. Interestingly, once symptoms could no long be kept private (eg, wind or diarrhoea), this could also act as a prompt for help-seeking. These findings add to previous studies relating to the effect of embarrassment on help-seeking,^10^ ^16^ ^30^ although interestingly embarrassment of physical examination or investigation was not a common barrier in this study. For the few participants in whom it was present, however, it could present a major barrier to prompt help-seeking. Our final theme identified culturally defined and implicit beliefs about the appropriate use of healthcare. Concern about ‘wasting the doctor's time’ is more common in the UK than in other comparable countries,^31^ and may be an important barrier to prompt help-seeking in publicly funded healthcare systems. The tension between wasting the doctor's time and raising a concern about health has been discussed previously,^32^ and a study of patients with lung cancer described how thresholds for healthcare-seeking reflected ‘shared societal norms of when professional care should be sought’.^33^

### Implications for practice and policy

Our findings have important implications for public health and primary care strategies to optimise early symptomatic diagnosis of CRC. The most novel finding relates to how the private nature of bowel symptoms can contribute to longer symptom appraisal and later presentation to healthcare. Once they are no longer ‘private’, they can also trigger help-seeking. Awareness campaigns should continue to aim to reduce the stigma of looking for changes in bowel movements and discussing bowel symptoms with family friends and health professionals.^34^ They also need to consider the mismatch between the expected and actual experience of cancer symptoms, particularly in relation to intermittent symptoms or clusters of non-specific, lower risk symptoms.^4^ However, awareness campaigns can exacerbate the tension between prompt help-seeking for common symptoms and concerns about wasting the doctor's time. Altering perceptions about the appropriate use of health services could have a beneficial effect on TTP, particularly in the UK and other countries with publicly funded healthcare.

In conclusion, the findings from this study provide fresh insights into how people appraise and act on their colorectal symptoms. We did not find any differences between people who progressed to a malignant or benign diagnosis. The findings could underpin novel approaches to community awareness campaigns.
